# 7^th^ Brazilian Guideline of Arterial Hypertension: Chapter 8 - Hypertension and Associated Clinical Conditions

**DOI:** 10.5935/abc.20160158

**Published:** 2016-09

**Authors:** MVB Malachias, C Amodeo, RB Paula, AC Cordeiro Júnior, LBNC Magalhães, LC Bodanese

## Diabetes mellitus

The association of AH and DM doubles the CV risk and has increased the AH prevalence, which is related to the elevation in overweight and obesity rates, as well as the increase in the elderly population.^1^ The incidence of AH in type 1 diabetic patients increases from 5%, at the age of 10 years, to 33%, at the age of 20 years, and to 70%, at the age of 40 years.^2^ There is a strict relationship between the development of AH and the presence of albuminuria in that population.^3^ That increase in the AH incidence can reach 75-80% in patients with diabetic kidney disease.^4^ Approximately 40% of patients with a recent diagnosis of DM have AH.^5^ In approximately 50% of type 2 diabetic patients, AH occurs before the development of albuminuria. All diabetic hypertensives are at high CV risk. In addition to all complementary tests recommended for hypertensives, diabetic patients require the search for urine albumin excretion, fundoscopic eye exam and assessment of probable postural hypotension, which can characterize the presence of autonomic nervous system dysfunction.^6^

The BP targets to be achieved are still controversial. However, there is recent consensus on a BP target < 130/80 mm Hg. (GR: IIb; LE: B). For the NPT of AH in diabetic individuals, all recommendations expressed in Chapter 6 apply. The therapeutic choice should be based on drug efficacy and tolerability. Considering that all diabetic patients are at high CV risk, the initial treatment includes the association of at least two drugs of different classes.^7^ In diabetic hypertensives without nephropathy, all antihypertensive drugs can be used. In the presence of diabetic nephropathy, however, RAAS inhibitors are preferred.^8^ (GR: I; LE: A). Simultaneous use of ACEI and ARB should be avoided because of the risk of complications.^9,10^ Although worsening insulin resistance, BB are useful for BP control in diabetic patients, especially when used in combinations to treat hypertensives with CAD or HF.^11^

## Metabolic syndrome

Metabolic syndrome (MS) is characterized by the coexistence of CVRFs (low HDL-C, high triglycerides, AH and dysglycemia) either associated or not with central obesity (identified by the AC measure). The definitions of MS differ according to different entities. In 2009, those entities convened a task force to conciliate the different definitions of MS.^12^ The criteria are described in Chapter 4 about CV risk stratification. The presence of AH in MS increases global CV risk. The initial treatment is based on lifestyle changes in association or not with the use of drugs. Because nonpharmacological measures isolated do not control BP, pharmacological treatment is required whenever BP ≥ 140/90 mm Hg.^13^ There is no evidence of benefit in the use of antihypertensive agents for MS with normal BP levels. When dysglycemia is present, the preferred drugs to begin AH treatment in MS are RAAS blockers and CCB.^13-19^

## Coronary artery disease

The treatment of AH associated with CAD, which includes patients after myocardial infarction, with chest angina and myocardial revascularization, should preferably comprise BBs, ACEIs and ARBs, in addition to statins and acetylsalicylic acid. Beta-blockers have proven highly beneficial after AMI, especially within 2 years from the acute event.^20^ Similarly, ACEIs tested on that condition have also proven beneficial.^21,22^ In patients with chronic CAD and multiple RFs, such as AH, ACEIs have shown a favorable effect to reduce relevant clinical outcomes.^23^ (GR: I; LE: A). Regarding BP target, it is worth considering the likelihood of the J curve effect, demonstrated in different studies,^24-27^ in which the excessive BP reduction, mainly in DBP, can precipitate CV events in patients with obstructive CAD. Additional drugs to meet target BP (BP < 130/80 mm Hg) are CCBs and thiazide DIUs.^28^ (GR: IIa; LE: B).

## Stroke

Stroke is the most common manifestation of the vascular damage caused by AH. In transient ischemic attack (TIA), the neurologic deficit is solved in 24 hours, with no clinically detectable sequelae.

### Pharmacological treatment of AH in the patient with previous stroke

Chronically, the effective antihypertensive therapy, maintaining BP < 130/80 mm Hg, has played a decisive role in the secondary prevention of all types of stroke and TIA.^29-35^ (GR: IIa; LE: B). As long as BP is reduced, any antihypertensive drug can be used.^20,36,37^ There is no clinical evidence allowing a definitive conclusion about the preferential use of ARBs as compared to other antihypertensive drugs for the secondary prevention of stroke.^34,35^ There is currently no evidence showing the effectiveness of beginning antihypertensive therapy for SBP < 140 mm Hg for patients with a previous stroke. (GR: III; LE: B).

## Chronic kidney disease

For patients with that disease, BP reduction is the most effective measure to reduce CV risk and to slow kidney damage progression, regardless of the antihypertensive drug used.^38,39^ (GR: I; LE: A). Special attention should be paid to patients with high albuminuria, which determines the unfavorable course of kidney disease^40^ and increases CV risk.^41^ (GR: IIa; LE: A). Elderly patients with renovascular disease, CAD and risk for postural hypotension often require customization of the antihypertensive treatment.^40^ (GR: IIa; LE: C). Usually, BP levels < 130/80 mm Hg are recommended, especially for those with albuminuria > 30 mg/g of creatinine and diabetic patients.^42,43^ In such patients, maintaining BP < 130/80 mm Hg reduces albuminuria and the risk for stroke, but there is no evidence that it decreases CV events and mortality.^44,45^ (GR: IIa; LE: A). However, it is controversial whether BP reduction to those levels is associated with better CKD course and with a reduction in mortality.^7,46,47^ (GE: IIb; LE: B). The present guideline suggests the adoption of BP targets shown in Chart 1.

**Chart 1:** Blood pressure targets for patients on conservative treatment, according to kidney disease etiology and albuminuria

	ALBUMINURIA < 30 mg/24 hours	ALBUMINURIA > 30 mg/24 hours
Non-diabetic CKD	< 140/90 mm Hg	< 130/80 mm Hg
Preferential drug	Any	ACEI or ARB
Diabetic CKD	<130/80 mm Hg	<130/80 mm Hg
Preferential drug	Any	ACEI or ARB

CKD: chronic kidney disease; ACEI: angiotensin-converting-enzyme inhibitor; ARB: angiotensin receptor blocker.

### Choice of antihypertensive drug: stage 1 to 5 chronic kidney disease on conservative treatment

Thiazide DIUs are recommended, because they are effective in stages 1, 2 and 3 CKD, while loop DIUs are recommended for stages 4 and 5 CKD. That drug class reduces CV morbidity and mortality,^48,49^ being considered the choice for association in CKD.^38,49,50^ (GR: I; NE: A). The ACEIs and ARBs are widely used for CKD, being effective for AH control and albuminuria reduction.^51-55^ (GR: I; LE: A). Regarding direct renin inhibitors and mineralocorticoid receptor antagonists, both with an antiproteinuric action, there is no evidence for their use in clinical practice.^56-58^ The risk of hyperpotassemia should be considered, especially with the latter. The double RAAS block is controversial. The combination of ACEI with ARB^59,60^ or of a renin inhibitor with ACEI or ARB^10^ has resulted in more acute kidney damage and hyperpotassemia, leading to a ban on that strategy from nephrological practice. (GR: I; LE: A). However, in a recent study on adult polycystic kidney disease^61^ and a meta-analysis on diabetic patients with CKD,^62^ the association of IECA and ARB has delayed the course of nephropathy without causing severe hyperpotassemia and acute kidney damage. (GR: IIb; LE: B). However, the double RAAS block remains contraindicated. (GR: I; LE: A). The CCBs are effective, especially for combined use with ACEI or ARB, being associated with a reduction in CV events.^63,64^ Other options include BBs, adrenergic inhibitors of central action, and, occasionally, direct acting vasodilators, such as minoxidil and hydralazine.

### Approach to stage 5 chronic kidney disease on kidney replacement therapy

Most studies on AH in patients with CKD undergoing dialysis is based on measuring pre-dialysis BP levels. However, BP obtained in that way is known to have large variability, in addition to being usually overestimated, as it is underestimated when obtained after dialysis.^65,66^ In those patients, BP should be preferably measured outside the dialysis centers, in the interdialytic intervals.^67^ (GR: IIa; LE: B). Home BP measures are more reproducible than those obtained before and after dialysis, have a fair association with both 44-hour ABPM and CV prognosis in patients undergoing dialysis.^68-70^ (GR: IIa; LE: B). In addition, a randomized study has shown that therapeutic decisions based on HBPM associate with better interdialytic BP control assessed with 24-hour ABPM as compared to pre-dialysis BP measurement.^71^ Regarding ABPM, it is worth noting that, although the 44-hour long exam is considered gold-standard for assessment of hemodialysis patients, its technical difficulties favor the use of 24-hour ABPM and home BP measurements.

The association between BP and mortality in patients with CKD undergoing dialysis has a "U" distribution for SBP and DBP, thus, both elevated and reduced levels relate to bad prognosis.^70^ (GR: IIa; LE: B). There are not enough studies to support with satisfactory level of evidence the diagnosis of AH in patients undergoing dialysis; however, the most accepted pre- and post-hemodialysis BP levels for that purpose are ≥ 140/90 mm Hg and ≥ 130/80 mm Hg, respectively.^70,71^ (GR: IIa; LE: C). A study with 326 hemodialysis patients has associated better prognosis with mean SBP levels between 120 and 130 mm Hg, in HBPM, and between 110 and 120 mm Hg, in ABPM.^68^ (GR: IIb; LE: B).

Because, in that population, hypervolemia plays a major role in AH etiology, the therapeutic management should consider that variable, focusing the treatment on gradual control of "dry weight", via salt and water restriction, in addition to promoting adequate ultrafiltration during hemodialysis sessions.^71-75^ (GR: IIa, LE: B). The choice of antihypertensive drugs should be individualized and based on characteristics, such as comorbidities, and drug's cardioprotective effect, intra- and interdialytic pharmacokinetic characteristics, and side effects.^71,72^ (GR: IIa; LE: C).

In kidney-transplanted patients, CCBs are a good option for AH treatment, because they are effective antihypertensive agents that antagonize arteriolar vasoconstriction caused by cyclosporine.^76^ The RAAS blockers can improve the transplant outcome in patients with increased urine albumin excretion. Diuretics, BBs, central action sympatholytic drugs and vasodilators can be used based on clinical judgement.^77,78^
